# Supplementary material for: Model misspecification, measurement error, and apparent supralinearity in the concentration-response relationship between PM2.5 and mortality
Source: PLoS One. 2024 May 23;19(5):e0303640. doi: 10.1371/journal.pone.0303640 (PMC11115258; doi:10.1371/journal.pone.0303640)
Supplement: S1 Table — (DOCX) [file pone.0303640.s001.docx]

| **Std Err** | **Distribution Truncation (± µg/m^3^)** | **0.005** | **0.01** | **0.02** | **0.03** | **Average** |
| --- | --- | --- | --- | --- | --- | --- |
| 0.05 | 4 | 7 | 2 | 2 | 2 | 3.3 |
|  | 5 | 10 | 8 | 9 | 7 | 8.5 |
|  | 6 | 6 | 2 | 0 | 0 | 2.0 |
| 0.1 | 4 | 42 | 35 | 33 | 30 | 35.0 |
|  | 5 | 44 | 39 | 40 | 37 | 40.0 |
|  | 6 | 52 | 40 | 34 | 34 | 40.0 |
| 0.2 | 4 | 26 | 12 | 9 | 8 | 13.8 |
|  | 5 | 49 | 29 | 23 | 21 | 30.5 |
|  | 6 | 56 | 46 | 44 | 42 | 47.0 |
| Average |  | 32.4 | 23.7 | 21.6 | 20.1 | 24.4 |
