# Supplementary material for: Model misspecification, measurement error, and apparent supralinearity in the concentration-response relationship between PM2.5 and mortality
Source: PLoS One. 2024 May 23;19(5):e0303640. doi: 10.1371/journal.pone.0303640 (PMC11115258; doi:10.1371/journal.pone.0303640)
Supplement: S2 Table — (DOCX) [file pone.0303640.s002.docx]

| **Std Err** | **Distribution Truncation (± µg/m^3^)** | **0.005** | **0.01** | **0.02** | **0.03** | **Average** |
| --- | --- | --- | --- | --- | --- | --- |
| 0.05 | 4 | 0 | 0 | 0 | 0 | 0 |
|  | 5 | 0 | 0 | 0 | 0 | 0 |
|  | 6 | 1 | 0 | 0 | 0 | 0.3 |
| 0.1 | 4 | 0 | 0 | 0 | 0 | 0 |
|  | 5 | 0 | 0 | 0 | 0 | 0 |
|  | 6 | 1 | 0 | 0 | 0 | 0.3 |
| 0.2 | 4 | 0 | 0 | 0 | 0 | 0 |
|  | 5 | 0 | 0 | 0 | 0 | 0 |
|  | 6 | 0 | 0 | 0 | 0 | 0 |
| Average |  | 0.2 | 0 | 0 | 0 | 0.1 |
