# Supplementary material for: Model misspecification, measurement error, and apparent supralinearity in the concentration-response relationship between PM2.5 and mortality
Source: PLoS One. 2024 May 23;19(5):e0303640. doi: 10.1371/journal.pone.0303640 (PMC11115258; doi:10.1371/journal.pone.0303640)
Supplement: S3 Table — (DOCX) [file pone.0303640.s003.docx]

| **Scale** | **Count Supralinearity**  **(Slope Estimation)** | **Count diffAICs > 2** |
| --- | --- | --- |
| 1 | 3 | 10 |
| 3 | 8 | 10 |
| 5 | 6 | 7 |
| Linear | 3 | 0 |
