# Supplementary material for: Model misspecification, measurement error, and apparent supralinearity in the concentration-response relationship between PM2.5 and mortality
Source: PLoS One. 2024 May 23;19(5):e0303640. doi: 10.1371/journal.pone.0303640 (PMC11115258; doi:10.1371/journal.pone.0303640)
Supplement: S5 Table — (DOCX) [file pone.0303640.s005.docx]

| **Relative Toxicity (City 1/City 100)** | **% of PM_2.5_ City 1** | **% of PM_2.5_ City 100** | **Count Supralinearity (Slope Estimation)** | **Count diffAICs > 2** |
| --- | --- | --- | --- | --- |
| 0.43 | 30 | 70 | 0 | 0 |
| 0.53 | 40 | 75 | 0 | 0 |
| 0.83 | 25 | 30 | 3 | 0 |
| 1.00 | 50 | 50 | 3 | 0 |
| 1.00 | 100 | 100 | 1 | 0 |
| 1.00 | 150 | 150 | 0 | 0 |
| 1.18 | 65 | 55 | 3 | 0 |
| 1.25 | 75 | 60 | 5 | 0 |
| 1.25 | 125 | 100 | 2 | 0 |
| 1.25 | 150 | 120 | 1 | 1 |
| 1.36 | 75 | 55 | 5 | 0 |
| 1.36 | 150 | 110 | 5 | 1 |
| 1.38 | 55 | 40 | 5 | 0 |
| 1.46 | 95 | 65 | 6 | 0 |
| 1.50 | 75 | 50 | 6 | 0 |
| 1.50 | 90 | 60 | 6 | 0 |
| 1.50 | 150 | 100 | 7 | 1 |
| 1.56 | 125 | 80 | 7 | 1 |
| 1.63 | 65 | 40 | 6 | 0 |
| 1.67 | 25 | 15 | 3 | 0 |
| 1.67 | 75 | 45 | 6 | 0 |
| 1.67 | 125 | 75 | 9 | 2 |
| 1.67 | 150 | 90 | 9 | 3 |
| 1.75 | 70 | 40 | 6 | 0 |
| 1.79 | 125 | 70 | 9 | 2 |
| 1.88 | 75 | 40 | 6 | 1 |
| 1.88 | 150 | 80 | 9 | 5 |
| 2.00 | 70 | 35 | 6 | 0 |
| 2.00 | 80 | 40 | 9 | 1 |
| 2.13 | 85 | 40 | 9 | 1 |
| 2.14 | 75 | 35 | 6 | 0 |
| 2.29 | 80 | 35 | 8 | 1 |
| 2.33 | 70 | 30 | 7 | 0 |
| 2.50 | 50 | 20 | 4 | 0 |
| 2.50 | 75 | 30 | 6 | 0 |
| 2.71 | 95 | 35 | 3 | 2 |
| 2.83 | 85 | 30 | 2 | 0 |
| 3.00 | 30 | 10 | 1 | 0 |
| 3.00 | 75 | 25 | 1 | 0 |
| 3.00 | 150 | 50 | 0 | 7 |
| 3.13 | 125 | 40 | 0 | 4 |
| 6.00 | 150 | 25 | 0 | 9 |
| 6.25 | 125 | 20 | 0 | 7 |
| 9.00 | 90 | 10 | 0 | 3 |
