# Supplementary material for: Model misspecification, measurement error, and apparent supralinearity in the concentration-response relationship between PM2.5 and mortality
Source: PLoS One. 2024 May 23;19(5):e0303640. doi: 10.1371/journal.pone.0303640 (PMC11115258; doi:10.1371/journal.pone.0303640)
Supplement: Attachment — Submitted filename: Response_to_Reviewers.docx [file pone.0303640.s006.docx]

Thank you for the opportunity to revise our manuscript, and thank you to both reviewers for the helpful comments. Below we respond to each point raised by the reviewers, and describe the edits we made in response.

Reviewer #1:

1. *To build their simulated cohorts, the authors say that "The baseline mortality rate for the simulated individuals in our cohort was calculated using cohort life tables compiled by the US Social Security Administration (Bell and Miller 2005, Table 7). These life tables give the probability of mortality at each age based on birth year and sex, with birth year reported in 10-year increments from 1900 to 2100". However, total mortality includes natural mortality and mortality due to other causes (car accidents, natural disasters, homicides and so on) which may not be influenced by PM2.5 exposure: thus, if they used total mortality, they should state this clearly and acknowledge this fact as a limitation of the study (or, better, run again the simulation using natural mortality rates).*

It is true that the baseline mortality in our simulations is for all-cause mortality, and not just mortality due to causes that may be influenced by PM2.5 exposure (e.g., COPD). Replacing all-cause mortality with natural mortality would reduce the baseline mortality in our simulations, but would not affect the simulation results, which measure the relative risk due to increased (simulated) PM2.5 exposure, and thus are not dependent on the baseline starting point.

However, we agree the types of mortality included in our baseline should be clearly stated, and that we should emphasize our simulation results cannot be used to infer anything about absolute risk. We have added text in Section 2.1 to address this point.

2. *Another issue is that the simulated cohorts are composed using only males data: as, apparently, mortality assumed to be related to air pollution levels has been shown, in epidemiological studies, to be possibly different between males and females, the authors should discuss this issue.*

We agree that it is important to emphasize that our simulations cannot be used to understand differences in relative risk based on sex. We have added text and a citation in Section 2.1 acknowledging that there could be differences in the effect of PM2.5 on males and females, and emphasizing that our simulation results cannot be used to understand the relative risk of PM2.5 exposure based on sex.

Reviewer #2: 

*1. A lot of the results rely on the test of supralinearity and I am not sure it has been explained enough.
1.1. In particular, the thresholds for "significance" (1.2) is not really justified and does not seem to rely on any asymptotic result. It would be useful to have an idea of its power in different situations.*

This comment has made us realize that our description of the slope ratio test needs to be revised. We conceived of this test as a more systematic version of the “eyeball” test other papers describing supralinearity have used, rather than a statistical test. For example, Liu et al. (2019) simply plot their estimated C-R curves, and note:

“The slopes for both curves were steeper at concentrations lower than 40 μg per cubic meter for PM10 and lower than 20 μg per cubic meter for PM2.5. The slopes seemed to flatten at high ranges. In addition, positive associations were still detectable at levels below most global and regional air-quality guidelines or standards.”

We have revised our description of the slope ratio test to clarify our goals. We also agree that a statistical test could be developed, although we think it would be a large undertaking worth a paper by itself. We describe our thinking on this point in more detail below.

*1.2. An alternative to this test could a simpler Wald test based on second derivative of the C-R function. Estimating the C-R with B-splines, one can show that a concave function would mean negative second differences between the B-Spline coefficients (say d2 beta, see e.g.* [*https://doi.org/10.1007/s11222-013-9448-7*](https://urldefense.com/v3/__https:/doi.org/10.1007/s11222-013-9448-7__;!!O7V3aRRsHkZJLA!COoMgxo8IGUTG_pbMs2sfXg3CSgJbmkA0C0XuoPBfviS1HQqkPlsq1NhHMUuf-70FNDuLsH4xRfdeqqwY50$)*). From there a Wald test testing H0: d2 beta = 0 (linearity) vs H1 d2 beta < 0 (supralinearity) can do the trick.*

We agree that a simple statistical test for supralinearity would be very useful to researchers. We have invested some time looking into this, and we think it would be a significant undertaking worth a paper by itself.

One issue with developing a formal statistical test is that there is no formal definition of “supralinearity” in the literature. Instead, most researchers describe a curve as “supralinear” if it is increasing more rapidly at lower concentrations of PM2.5.

For example, consider the estimated C-R function from one simulation from one of our “toxic PM” scenarios where 50% of PM2.5 is toxic at the lowest concentration, and 20% of PM2.5 is toxic at the highest concentration.

Most researchers would categorize this C-R function as supralinear, because incremental risk is higher at the lowest exposure levels than at the highest exposure levels. However, this C-R function is not perfectly concave, so it would not pass the second derivative test suggested here. We have also looked into other tests, such as positive and declining first derivatives across PM2.5 exposure, but again, this particular function would not pass that test. It should be possible to develop a test in which the first or second derivatives *mostly* match the conditions we lay out, but this would require a significant amount of research, which is why we think a formal statistical test for supralinearity deserves its own manuscript.

We have updated the manuscript to drop references to “concavity” when describing supralinearity as this can be misleading. We have also added some discussion around Figure 4 (described below) on the challenges in coming up with a formal test for supralinearity.

*1.3 In addition to the test, I think it would useful to display the estimate C-R curves for many scenarios. It would provide an idea of to which extent the scenario creates supralinearity where there is none.*

We agree, and have provided 15 examples of different C-R curves in a new figure (Figure 4).

*2. Performing the simulations is interesting to understand how models behave in various situations, but I feel the models applied here do not really reflect the current practice in long-term PM2.5 studies. My understanding from the study is that Cox models are applied with no confounders or stratification. But in practice, many studies will stratify by location (assessment center, city, ...) or include some proxy as a confounder in the model. In this case, would the apparent supralinearity persist nonetheless?*

We agree that most studies on the relationship between PM2.5 and mortality stratify by location and include controls for some demographic characteristics that proxy for confounders (age, sex, etc.). Since our simulations assume no differences between individuals beyond PM exposure and no differences between cities beyond PM levels, our results are equivalent to those that would apply to a single strata and demographic group. In theory, our simulations should produce the same results with more demographic groups and non-PM city-specific mortality effects if we correctly stratify and control for demographics.

However, in practice we found that including additional demographic groups and non-PM city-specific mortality effects made it difficult to determine the shape of the C-R function, with large amounts of variation in the C-R function shapes across simulations with the same inputs, unless the populations we simulated were extremely large. Thus, we opted to simplify the simulation population to reduce non-PM related variation, which allowed us to use smaller population sizes and undertake a more comprehensive set of simulations.

This does represent a limitation of our study. We describe this point in the discussion.

*3. About the PM2.5 composition scenarios
3.1. I would be curious to see some scenario where the differences in PM2.5 toxicity is independent from the total PM2.5 mass. The idea behind the PM2.5 composition studies is that, for fixed PM2.5 levels there are probably different vulnerability because of the relative toxicity of the local mix.*

If the differences in PM2.5 toxicity are independent from total PM2.5 mass, this will be equivalent to a classic measurement error case, in which “true” (toxic) PM2.5 differs randomly from the “measured” (total) PM2.5. In this case we will see an attenuation of the estimated hazard ratio, but no estimated supralinearity. To get supralinearity we need non-independence between PM2.5 mass and PM2.5 toxicity.

*3.2.	Considering the "proportion of toxicity" is fine as a simplified model, but it could be acknowledged that potentially all PM2.5 component are toxic (even the natural ones such as desert dust), they just are toxic to different degrees.*

We agree, and have added text and a citation to indicate that potentially all PM2.5 components are toxic to some degree. We also note that our simulations results depend on the relative toxicity of PM2.5 concentrations, not the simplifying assumption that some PM2.5 is non-toxic.

*4. About the measurement error scenario:
4.1. You could argue that* *this one is becoming important with the advent of reconstructed exposure datasets (e.g.* [*https://doi.org/10.3390/rs12223803*](https://urldefense.com/v3/__https:/doi.org/10.3390/rs12223803__;!!O7V3aRRsHkZJLA!COoMgxo8IGUTG_pbMs2sfXg3CSgJbmkA0C0XuoPBfviS1HQqkPlsq1NhHMUuf-70FNDuLsH4xRfdfe_yN_A$)*), that probably display higher error than a monitoring station.*

This is a good point. We have added text and the provided citation to the discussion addressing this.

*4.2. I would expect that a common error would actually be a systematic bias. Would the results hold in such a case?*

Whether our results would hold if measurement error was systematic rather than random will depend on what sort of systematic bias exists. If all air quality monitors are systematically overestimating (underestimating) PM this would bias the estimated hazard ratio downward (upward), but would not induce supralinearity. However, some other types of systematic error could induce supralinearity. For example, systematically overestimating PM2.5 exposure by a constant percentage could also induce apparent supralinearity. We have added a sentence to the conclusion describing this scenario.

*# Minor
- The paper focuses on long-term studies, but note that short-term studies also suggest potential nonlinearities (e.g.* [*https://doi.org/10.1056/NEJMoa1817364*](https://urldefense.com/v3/__https:/doi.org/10.1056/NEJMoa1817364__;!!O7V3aRRsHkZJLA!COoMgxo8IGUTG_pbMs2sfXg3CSgJbmkA0C0XuoPBfviS1HQqkPlsq1NhHMUuf-70FNDuLsH4xRfdm6eZNdI$)*).*

This is a good point. We have added text and the provided citation to the discussion addressing this.

*- p.7 line 1: a simpler way to explain this is sampling from a Bernoulli with p being the probability of mortality*

We agree and have modified the text to describe the mortality draws in this way.

*- The model linking exposure to hazard ratio is not clear as the exposure appears nowhere in the equations.*

We have made two edits to clarify these formulas. First, in section 2.3 we have edited the formula to clarify the role of PM2.5 exposure. Second, we have deleted the formula in section 2.5, and refer readers back to Section 2.3.

*- I am not sure I understand what the authors mean when they mention that the true C-R is not perfectly linear (p. 13 l. 4). Aren't we trying to estimate the hazard ratio h, which represents a linear relationship in the simulations?*

In the set of simulations examining the effect of measurement error, the estimated C-R functions based on a linear C-R function turned out to be slightly sublinear due to random variation in mortality across cities. Thus, our worry was that shifts towards a supralinear C-R function due to measurement error would not be detected, since the baseline case was sublinear rather than linear due to random variation in the cohorts we generated. This is the reason the slope ratio test for these simulations was applied relative to the C-R function with no measurement error (i.e., we test whether measurement error shifts the apparent C-R function in a supralinear direction relative to the “true” C-R function).

We have added some text to Section 2.6 to explain this more clearly.
